# Knowledge, Attitudes, and Behaviors of Viral Hepatitis Among Recent African Immigrants in the United States: A Community Based Participatory Research Qualitative Study

**DOI:** 10.3389/fpubh.2020.00025

**Published:** 2020-03-06

**Authors:** Essa A. Mohamed, Nasra H. Giama, Hassan M. Shaleh, Linda Kerandi, Abdul M. Oseini, Hager Ahmed Mohammed, Henry Kerandi, Loretta K. Allotey, Ibrahim A. Waaeys, Hamdi A. Ali, Hawa M. Ali, Safra A. Mohamed, Ju Dong Yang, Wudneh O. Gaga, Lily L. Tamire, Awol Windissa, Christi A. Patten, Joyce E. Balls-Berry, Lewis R. Roberts

**Affiliations:** ^1^Division of Gastroenterology and Hepatology, Mayo Clinic, Rochester, MN, United States; ^2^Urgent Care and Clinic, Midpoint Medical Clinic, Brooklyn Park, MN, United States; ^3^Division of Gastroenterology, Virginia Commonwealth University, Richmond, VA, United States; ^4^Medical School, University of Minnesota, Rochester, MN, United States; ^5^Somali Health Advisory Committee, Rochester, MN, United States; ^6^St Olaf College, Northfield, MN, United States; ^7^Transplant Hepatology, Cedars-Sinai Medical Center, Los Angeles, CA, United States; ^8^St. George Ethiopian Tewahido Orthodox Church, Rochester, MN, United States; ^9^Pillsbury United Communities, Minneapolis, MN, United States; ^10^Behavioral Health Research Program, Mayo Clinic, Rochester, MN, United States; ^11^Department of Epidemiology, Mayo Clinic, Rochester, MN, United States

**Keywords:** immigrant health, community based research, liver cancer disparities, African, viral hepatitis

## Abstract

**Background:** In the United States, hepatocellular carcinoma is the ninth leading cause of cancer mortality. Hepatocellular carcinoma disproportionately affects individuals of African ancestry with the rates being higher amongst individuals of foreign-born African ancestry. This study explored knowledge, attitudes, and behaviors toward viral hepatitis transmission, screening, and vaccination among recent African immigrants in Minnesota and identify ways to improve early detection and screening methods.

**Methods:** A community based participatory research (CBPR) team with minority researchers and community members sought to gain insight on persons of African Ancestry knowledge, attitudes, and behaviors related to viral hepatitis by conducting a qualitative research study. The CBPR team developed a focus group moderator's guide with semi-structured questions related to transmission, screening, and vaccination of viral hepatitis. We conducted seven focus groups using bilingual, bicultural moderators with participants from local Ethiopian, Liberian and Kenyan communities from August 10th, 2014 to October 11th, 2014. Focus groups were audio recorded and transcribed. The CBPR team categorized the data into themes and subthemes with consensus using traditional content analysis.

**Results:** Community partners recruited 63 participants with a majority identifying as male (51%). Participants lacked knowledge of viral hepatitis screening, vaccination, and treatment. Participants were aware of some behaviors that increased risk of acquisition of hepatitis. Participants endorsed a strategy of developing and delivering educational materials for African immigrants. Moreover, access to care and cultural awareness were mentioned as pivotal for prevention and treatment of viral hepatitis.

**Conclusions:** Findings from this pilot study provide insight on areas of research focus. Having a research team consisting of members from the community helped to increase trust and foster an understanding of shared community values. Information from this study provides evidence to support the development culturally appropriate strategies to address disparities in viral hepatitis in these communities.

## Background

Hepatocellular carcinoma (HCC) is the most common primary cancer of the liver (1) and has the sixth highest cancer incidence worldwide (2). The World Health Organization estimates that HCC incidence will continue to rise until 2030 (3). The most common risk factors for the development of HCC are chronic hepatitis B virus (HBV) and hepatitis C virus (HCV) infections (4). Due to the high prevalence of viral hepatitis in low- and middle-income countries in Asia and Africa, HCC incidence is highest in these regions (5–9).

The 2012 Cancer Report by the Minnesota Department of Health reported the incidence and mortality rates due to liver cancer among the Black population were higher than their White counterparts (10). African immigration to Minnesota is the third highest by percentage of state population (11).

The incidence of HCC in the African continent is increasing due to rising numbers of viral hepatitis infections (12–17). These findings suggest that immigrant African populations from regions endemic for viral hepatitis are at substantial risk of developing HCC. According to the US Census, Minnesota's four largest African populations are from Somalia, Ethiopia, Liberia, and Kenya (11). Viral hepatitis disproportionately affects sub-Saharan Africans (15, 16, 18–21) and these individuals are emigrating from countries where childhood HBV vaccination has only recently been implemented (22). Therefore, we speculate that this community may be a major contributor to the increased burden of viral hepatitis and liver cancer in the state.

A recent hospital-based study examined HBV and HCV infection among Somalis seen at Mayo Clinic and compared them to non-Somali Olmsted County residents (90% of whom were White). Among Somalis, the adjusted-frequency of HBsAg positivity (chronic HBV) was 10-fold higher than in non-Somalis, HBcAb positivity (prior HBV exposure) was 5-folder higher than in non-Somalis and anti-HCV positivity (chronic HCV) was 3-fold higher than in non-Somalis (23). Subsequently, a prospective study screening 850 Somali community participants from 2010 to 2014 found a 17% frequency of chronic HBV infection, 30% frequency of past exposure to HBV and 9% frequency of chronic HCV infection (Unpublished Data).

The purpose of this study was to identify societal and cultural barriers to the prevention, screening, and treatment of viral hepatitis in African immigrants and refugees in the United States. We conducted a qualitative pilot study to assess the knowledge, attitudes, and behaviors of African immigrants (AI) to provide pilot data for the eventual development a community-engaged intervention to (a) increase awareness and (b) reduce the burden of viral hepatitis and HCC in these communities.

## Methods

### Community-Based Participatory Research (CBPR) Study Design

A CBPR partnership formed in 2013, called Somali Health Advisory Council (SHAC), between an academic medical center and three AI communities in Minnesota. The research team included members from Mayo Clinic Rochester and AIs from Ethiopia, Liberia, and Kenya. The Mayo Clinic Institutional Review Board approved the study. The team jointly designed the study and selected a qualitative research approach using focus groups to address the specific aims.

### Participants Recruitment

The SHAC designed the recruitment process with the research team. We used purposive sampling (24) and planned with the goal of collecting data until we reached saturation. Recruitment focused on the three AI communities (25). The community-engaged recruitment included flyers, word-of-mouth (snowball sampling) and telephone calls with outreach to religious organizations, local businesses, community-based centers, organizations, and clinics. Eligibility criteria were individuals who self-identified AI and ≥18 years old.

### Focus Group Procedures

Focus groups allowed for observations of non-verbal behaviors and interactions as well as group dynamics and to elicit shared cultural meanings (26). The focus group moderator guide, contained semi-structured questions and probes, was adapted from a study conducted in Pakistan by community members from the SHAC (27). The SHAC community members and leaders were interested in increasing health and wellness in the AI by using biomedical research to increase health awareness in the community. AI bicultural and bilingual moderators and note-takers were recruited and trained to complete the data collection. A team member with expertise in qualitative research (NG) trained focus group moderators and note-takers. We used a three-step process to train our moderators and note-takers: (a) an 8-h didactic course, (b) an 8-h focus group technique practice using mock focus groups, and (c) implementation of two actual focus groups with observation and feedback from trainers. To increase the reliability of the findings we conducted more than one focus group for each of the three immigrant communities (25). The groups were not separated by gender as it was thought from a cultural perspective that participants would be sharing in mixed gender groups.

### Focus Group Data Collection

Several study participants had low levels of English proficiency. To ensure participants understood the risks and benefits of our study, the purpose of the study was stated in their native language by an interpreter. Participants provided written informed consent and received a copy of the informed consent form. Participants received a $20 gift certificate and a light meal for their time.

Prior to starting the focus group discussions, facilitators (a) introduced themselves, (b) outlined the structure of the focus group, (c) instructed in the importance of confidentiality, and (d) collected selected sociodemographic information through a brief survey.

Focus groups facilitated in native language were transcribed verbatim and translated into English by WG and LT. Data were de-identified and unique IDs given to each participant and moderator. Participants were asked general questions pertaining to beliefs of health which progressed to specific questions pertaining to viral hepatitis and liver cancer (Appendix A). The focus groups were audio recorded. Audio files of the interviews were transcribed into English (EM, HS, and NG).

### Qualitative Analysis

Members (EM, NG, HS, and IW) from the research team independently conducted initial review and coded transcripts. The research team used a strategic validation process to ensure that inductive thematic saturation was reached. Preliminary thematic groups were generated and were linked to interview texts. Emerging themes were discussed and presented at team meetings. During these meetings, discordant codes were discussed until consensus was reached. Differences between the three community groups and by gender were explored.

## Results

### Participants

The digitally recorded focus groups included 5–12 participants and were ~90 min in duration (range 70–110 min). In total, seven focus groups were conducted (*n* = 63) from the three AI communities. Participants self-identified as Ethiopian-Amhara, Ethiopian-Oromo, Liberian, or Kenyan. Males constituted about half [32 (51%)] and the mean age ± SD was 46.6 ± 19.4 years. Eleven percent of participants reported not having medical insurance and the mean ± SD years lived in the US was 12.2 ± 6 years. Approximately a third had completed college and 16% had postgraduate training (Table 1).

**Table 1:** Demographics of focus group participants.

**Items**	***n***	**(%)**
Age (Mean ± SD)	46.6 ± 19.4	
Gender identity		
Male	32	51
Female	31	49
Education		
Less than Undergraduate	32	51
Undergraduate	21	33
Graduate	10	16
Average years residing in the US	12.2 ± 6	
No medical insurance	7	11

### Themes

Three major themes emerged from analysis of the data: (a) lack of knowledge of viral hepatitis; (b) cultural and societal barriers associated with viral hepatitis; and (c) need for culturally relevant health education. There were no differences noted between communities or by gender, thus the results are consistent for the three communities.

## Theme 1: Limited Knowledge and Awareness of Viral Hepatitis

### Knowledge

The knowledge of viral hepatitis across indicated limited understanding and awareness of the infection (Table 2). Most participants did not know the modes of transmission, prevention, and development of liver disease. In addition, participants were not aware of the different types of viral hepatitis and did not have access to information pertaining to these different types of infection. Most participants agreed viral hepatitis was not promoted in public health efforts to the same degree as other infections (i.e., HIV or malaria). A participant in the medical field stated she did not know anything about viral hepatitis and the different types of viral hepatitis until she did her nursing assistant training.

**Table 2:** Limited knowledge and awareness of viral hepatitis.

**Themes**	**Selected quotations**
Knowledge	“*I don't think any community is fully familiar with knowledge about hepatitis, whether it's A, B, C…”*
	“*I discovered that there are different types of hepatitis and that it can be transmitted. We just didn't have access to information about the different kinds”*
	“*We have a lot more information on HIV than hepatitis”*
	“*I'm a nurse now but I first encountered hepatitis B while I was studying to take the nursing assistance course. If people don't have those types of experiences it's unlikely they know anything about any hepatitis”*
	“*What's hepatitis? I actually, I don't know what it is?”*
	“*What is the function of the liver”*
	“*Can someone explain what liver disease is…we might not know what it is”*

When asked, “What is viral hepatitis?” several members mentioned they did not know what viral hepatitis is and needed more information. For instance, one participant mentioned, “*I don't think any community is fully familiar with knowledge about hepatitis, whether it's A, B, C.”* Moreover, several participants did not know the exact location or understand the basic function of the liver and had little understanding of the impact of liver disease on health.

### Progression of Liver Disease

When asked what cultural practices contribute to liver disease progression, most participants were aware that alcohol consumption led to long-term liver disease and associated complications. There was lack of awareness of secondary and tertiary prevent once a person is diagnosed with hepatitis by some participants. One participant maintained that “*there are different types of hepatitis and that it can be transmitted”* but “*didn't have access to information about the different kinds.”* Others mentioned a person might feel fatigued, have an enlarged stomach, and yellow coloration of skin and eyes. However, participants were unaware that most chronic viral hepatitis is asymptomatic and symptoms arise after the liver is compromised for several decades.

### Acquisition

Participants expressed a wide range of responses and evident confusion regarding modes of viral hepatitis acquisition. Several examples participants mentioned were sexual contact, bodily fluids, and blood transfusions. A participant stated viral hepatitis is contracted “*through kissing or through handshakes,”* while another stated it is “*transmitted through coughing”* (airborne). Others were convinced the acquisition and transmission viral hepatitis was through “*starvation,”* likening the round belly of a liver disease patient to a starving child's belly.

### Prevention

Participants discussed the role of cleanliness in reducing viral hepatitis transmission. Others further suggested changing dietary habits (i.e., reduce high fat intake, vegetarian diet, reducing alcohol consumption), utilizing clean syringes, reducing smoking habits and exercising regularly. However, some believed minimizing alcohol consumption reduced the risk of developing liver disease and viral hepatitis.

### Nomenclature and Health Literacy

Viral hepatitis is a complex set of infections not quite understood by the three communities. From the focus group discussions, participants lacked awareness of the scientific terms associated with the infection. Across the different communities, descriptive terms for viral hepatitis were related to symptoms of disease (i.e., Oromo: “*birdheellee”*; Amharic: “*bird's disease”*). In the Liberian group, the general term used to describe viral hepatitis was “*yellow jaundice.”* Most participants believed viral hepatitis was a disease not necessarily infectious; however, a small proportion felt it was infectious.

## Theme 2: Cultural and Societal Barriers to Access to Care

### Stigmatization

One constant subtheme across the different AI groups was the notion of stigmatization as a barrier to accessing care (Table 3). Inquiring about viral hepatitis- or liver cancer-status put a person at risk and their secret would be known to the community. Participants felt people would ostracize the infected individual because of the assumption that they have “*loose morals”* or have transgressed against others. Some participants mentioned that even when the severity of the disease is known, people still will not disclose their medical diagnosis, except to their immediate family members. Participants were concerned with the stigma associated with viral hepatitis, some participants stated they are not sure whether viral hepatitis is “*an issue”* with their community. Concerns of people fearing viral hepatitis will result in being poorly. Many described the poor treatment of patients living with HIV and the lack of cultural awareness of how to treat the disease.

**Table 3:** Cultural and societal barriers.

**Themes**	**Selected quotations**
Stigmatization	“*…our culture of ostracizing people who have the disease is…bad because you're a bad person or have loose morals. People are afraid to speak up…because we become the talk of the town…discourages people from coming out”*
	“*The doctor says I got stage 2 cancer. We don't do that. It's something back home your immediate family would know. Any illness or diagnosis, even if your next-door neighbor, they not know what you're going through. It's not common that you would just go and let them know your diagnosis”*
	“*There might be people with hepatitis in our communities, but just because, you know, you don't hear a lot about that [viral hepatitis]…”*
Barriers to accessing healthcare	“*They [elders] need someone who is speaking the dialect of the same community…person explain to everyone”*
	“*…we need somebody who speak the English to the doctor and the dialect that the person speaks”*
	“*…So when you find out that 30% of Kenyans living in Minnesota don't have papers, they know if they are caught they are on their way home. So where they work, they don't even get insurance because they know at the time they're caught, they're not going anywhere but going home”*
	“*The work I do gives me an idea of who has papers and who doesn't. And I can tell you that the percentage is higher than 30%”*
	“*…mistrust of hospitals and doctors by the public and that leads them [in reference to patients] to seek traditional healers”*
	“*What I do is take herbs from home. I took some…and it went down…the problem is getting things [herbal ingredients] from Africa is hard. There is a tree that grows in Liberia, in the eastern part and after the plant is used to make treatment for blood pressure”*
	“*Yellow jaundice [in reference to viral hepatitis] we use alternative medicine, herbs, we use rhubarb, herbs, to treat…in the process of treating with olden herbs”*

### Barrier to Accessing Healthcare

Participants spoke about several barriers to accessing medical care (Table 2). For example, language barrier was quite similar across the different groups. Several participants mentioned language barriers compromised their navigation through the medical system, the quality, and maintenance of care patients receive, thus translating into increased cases of preventable medical conditions. Participants mentioned they need someone who understands the medical language and is able to “*speak[ing] the dialect of the same community.”* This allows the translation of information effectively from English into their local dialect.

Numerous participants mentioned they do not have insurance because of their undocumented status in the US. Some fear if they seek medical care, there's a possibility of identification and deportation to their native country. This hinders access to care. As the discussion continued regarding how lack of documentation contributed to lack of medical access, another participant added that the “30% may be an underestimate” and that the true percentage of AI that are undocumented is much higher.

Another contributing factor to the reluctance to access healthcare is the lack of confidence in Western medical practice. For example, across the different AI communities many elderly participants preferred to go to traditional healers instead of medical doctors. When participants were asked ways to treat viral hepatitis, various participants mentioned cultural remedies used by their respective traditional healers. Many individuals trust the traditional healers and remedies and believe Western medicine does not improve but rather worsens symptoms.

## Theme 3: Health Education on Viral Hepatitis and Liver Cancer

### Community Health Education Needs

Participants expressed interest in learning more about viral hepatitis and its complications (Table 4). For example, the need to learn proper hand hygienic practices, vaccinations, and screening were topics of interest. Others discussed that partnerships for health education and promotion was most appropriate with community-based organizations and faith-based organizations (i.e., churches and mosques). Participants indicated the importance of having someone explain the pathophysiology of viral hepatitis. There was the suggestion that the CBPR team may host health screenings to evaluate the individual's health.

**Table 4:** Health education on viral hepatitis and liver cancer.

**Themes**	**Selected quotations**
Community Educational Need	“*Education. There are limits…but proper hand hygiene techniques could be taught. Vaccinations and screening should be available for everybody. There needs…community outreach programs for vaccination, education regarding transmission and screening”*
	“*…does a presentation at my church. So we had like a health day. He did not talk about a specific disease but like being healthy in general. More like exercise, eating healthy, you know? I don't know those kinds of things, not being specific to any kind of disease.”*
	“*…they did presentations at my church too. So there's a lot of Kenyans who are nurses…but then they focused on diabetes and cancer…”*
	“*I'll probably go if somebody took my blood pressure, heart rate, then explain that to me would help…”*
	“*…pastors should be educated so they can allow their church a 10-minute section to talk about this [in reference to viral hepatitis]…even if they can allow a 15-min program every Saturday or Sunday this will be very instrumental for change, for protection”*
Community resources	“*…there are special herbs that they [community] use that can treat [viral hepatitis]. The leaves—you boil it and you drink it warm”*
	“*…people are seeking traditional medicine and on the surface it seems like it works”*
	“*We need to know where the resources are. When my mother came, I didn't know where to start applying for insurance. I didn't encourage her to seek out vaccinations and health screening because it's expensive”*

Several participants mentioned the important role religious clergymen play in providing health education on viral hepatitis. A participant added it would help to have these religious clergymen become trained and educated on health issues. In one focus group a participant mentioned, “*…pastors should be educated so they can allow their church a 10-min section to talk about this [in reference to viral hepatitis]…even if they can allow a 15-mintute program every Saturday or Sunday this will be very instrumental for change, for protection.”* Across each AI focus groups participants mentioned the importance of involving influential community leaders like business owners and civic leaders in helping to provide awareness of viral hepatitis.

### Community Resources

Participants identified their preferred methods to receive health information. Several participants expressed their primary care physician would be their source of medical advice or they would seek tertiary care clinics for treatment (Table 3). On the contrary, some participants shared that community leaders, such as religious clergymen and traditional healers were their main sources of health education and healing.

However, participants mentioned they need assistance with determining where to obtain information on community resources and how to successfully acquire the information (i.e., applying for insurance, screening, vaccination, etc.). Lastly, several participants felt it is important for individuals to start discussing the infections freely to increase awareness and save lives are saved. More importantly, participants recognized that the need to address traditional medicine and use of herbs to treat hepatitis.

## Discussion

To our knowledge, this is the first qualitative research study examining knowledge, attitudes, and behaviors toward viral hepatitis amongst recent AIs in Minnesota. The findings illustrate that general knowledge of viral hepatitis transmission, prevention, and liver disease progression is poor among the three AI Minnesotan communities. The novel findings of this study were: (a) the willingness of participants to learn about these infections, the progression and development of disease, and optimal means of prevention and disease management, and (b) the open and welcoming attitude of the participants toward receiving screening for HBV and HCV in their community settings.

Various barriers to accessing care for viral hepatitis and liver disease within AI communities were described. One study assessing barriers to screening and vaccination amongst Korean-Americans identified lack of adequate health insurance, culturally competent providers, and a culturally specific notion of when one needs to access care as contributors to this disparity (28). This was supported from the findings in our study.

Overall, knowledge of viral hepatitis in the AI communities studied was quite minimal. Previous studies have shown that immigrant Asian and African communities have low levels of understanding of the causes and risk factors for liver cancer (28–32). In addition, our study indicates participants do not have an understanding of the different types of viral hepatitis, confirming a prior study assessing the knowledge of different viruses causing hepatitis (33). Furthermore, our focus group showed anecdotal associations of viral hepatitis to progression of liver disease as seen in other immigrant populations (34, 35). There was a consistent association of viral hepatitis to the notions of “yellow” coloration or “jaundice,” however, many of the participants did not know that chronic viral hepatitis may not present with symptoms (33). Culturally, participants did not view viral hepatitis as a serious health matter (35–37).

Our results indicate that participants had some level of understanding of the various modes of transmission of HBV and HCV. However, further education is needed to reinforce and enhance understanding of the different modes of transmission. This was evident as several participants mention that coughing and kissing were methods of contracting the infections. In addition, the lack of symptoms in the early stages of viral hepatitis appeared to be a barrier to seeking screening, as many participants expressed that people in their communities only seek health care for symptomatic illness. This supports previous research showing that lack of symptoms contributes to and increases the reluctance to seek screening (38–40).

In our study, access to medical care was linked to legal documentation status. According to the African Migration Insider, ~200,000 Kenyan nationals living in the US do not have proper documentation (41). This was reflected as a theme in our focus groups, with members reporting that about a third of the Kenyan population in Minnesota do not have proper documentation. This in turn became a barrier to seeking medical care due to fear of deportation. Other studies reported similar anecdotal fears of deportation amongst undocumented immigrants presenting at emergency rooms (42). Furthermore, it has been reported that undocumented immigrants use fewer medical services than their documented counterparts (43). State and national policies linking legal immigrant status to access to healthcare may therefore hinder access to medical care (44, 45). This lack of public support experienced by immigrant communities in this study stands in contradiction to earlier findings (46, 47), which show that early screening, vaccination, and treatment of immigrant communities for viral hepatitis is cost-effective.

Another subtheme that emerged as a barrier is language and understanding. This notion is quite consistent with previous research (48–51). Ways suggested to empower and enable the community were to utilize community-based organizations, such as churches and other faith communities as a source of health education (52).

The limitations of our study are that it is not generalizable to the larger United States AI populations. Recruitment for the focus groups was limited by the sample size, as this was a pilot study with insufficient resources to conduct additional focus group sessions to allow for a more comprehensive comparison between groups (i.e., age, gender, education, etc.). Moreover, the focus group participants were self-selected and may not represent the majority of the population. However, there were specific themes that were repetitive across the different focus group sessions.

## Conclusion

General knowledge of viral hepatitis transmission, prevention and liver disease progression is not well-understood among the AI communities in Minnesota. Similar studies were conducted in West Africa and Afro-Caribbean/Afro-Latinx populations. To our knowledge this is the first study to assess the perceptions and behaviors of viral hepatitis in an East African population, in particular from Kenya and Ethiopia. Although, Liberians are of West African descent, this study sought to determine the unique cultural and societal barriers to receiving treatment for viral hepatitis infection in this community. Currently, studies conducted in the United States on viral hepatitis infection fail to consider the disease implications for the AI community. A recent study by Ogumwobi et al. found that first generation African immigrants had limited knowledge the acquisition of hepatitis B (53). Thus, this impacts screening, treatment, and prevention efforts in these communities. Participants stated that they trust certain members of society when seeking health care. Participants maintained cultural taboos associated with sharing positive viral hepatitis status and requested free community-wide screening HBV and HCV. In addition, a culturally-sensitive program will be required to minimize the burden of disease and to address the fear of exposure after diagnosis, thus, facilitating active screening, treatment, and prevention of viral hepatitis in AI (32, 54).

## Data Availability Statement

All datasets generated for this study are included in the article/Supplementary Material.

## Ethics Statement

All study protocols, involved human participants, were approved by the Mayo Clinic Institutional Review Board and were in accordance with the ethical standards of the 1964 Helsinki Declaration and its later amendments or comparable ethical standards. This article does not contain any studies including animal subjects. All participants provided written informed consent and received a copy of the informed consent form.

## Author Contributions

EM, NG, and HS contributed to study concept and design, acquisition of data, data analysis, interpretation of data, and drafting of the manuscript. LK, HK, WG, LT, AO, HA, IW, LA, AW, HAA, HMA, and SM contributed to study concept and design, acquisition of data, interpretation of data, and critical revision of the manuscript. JY and CP contributed to study concept and design, interpretation of data, and critical revision of the manuscript. LR and JB-B contributed to study concept and design, interpretation of data, critical revision, and final approval of the manuscript. All authors contributed to manuscript revision, read, and approved the submitted version.

### Conflict of Interest

The authors declare that the research was conducted in the absence of any commercial or financial relationships that could be construed as a potential conflict of interest.

## Abbreviations

AI: African Immigrants
HBV: Hepatitis B Virus
HCV: Hepatitis C Virus
HCC: Hepatocellular Carcinoma
HBsAg: Hepatitis B surface-antigen
HBcAb: Hepatitis B core-antibody
anti-HCV: Hepatitis C antibody
CBPR: Community-Based Participatory Research.
