# Supplementary material for: Knowledge, Attitudes, and Behaviors of Viral Hepatitis Among Recent African Immigrants in the United States: A Community Based Participatory Research Qualitative Study
Source: Front Public Health. 2020 Mar 6;8:25. doi: 10.3389/fpubh.2020.00025 (PMC7067746; doi:10.3389/fpubh.2020.00025)
Supplement: Supplementary file 1 [file Data_Sheet_1.docx]

**Appendix A:** Topics on Focus Group Guide

- Describe and discuss your history of screening or lack of screening for viral hepatitis.
- Please describe your access to hepatitis B vaccine.
- Do you have health insurance? Describe the impact of health insurance on access to care for hepatitis.
- Describe the difference between Hepatitis B and Hepatitis C?
- How is viral hepatitis spread from person to person?
- What are common symptoms for viral hepatitis (jaundice, nausea, vomiting, loss of appetite)?
- Please explain if there are people diagnosed with viral hepatitis that may not have symptoms.
- Please describe the transmission of viral hepatitis (injection drug use, surgery, blood products, barbering, piercing, mother-to-child, water/food).
- Is there a cure for viral hepatitis (medical or self-curing)?
- Describe any dietary requirement for the treatment of viral hepatitis.
